# Proteomic analysis of differential protein expression by brain metastases of gynecological malignancies

**DOI:** 10.1007/s13577-012-0053-4

**Published:** 2013-03-16

**Authors:** Ayako Yoshida, Naoki Okamoto, Akiko Tozawa-Ono, Hirotaka Koizumi, Kazushige Kiguchi, Bunpei Ishizuka, Toshio Kumai, Nao Suzuki

**Affiliations:** 1 Department of Obstetrics and Gynecology, St. Marianna University School of Medicine, 2-16-1 Sugao, Miyamae-ku, Kawasaki, Kanagawa 216-8511 Japan; 2 Department of Diagnostic Pathology, St. Marianna University School of Medicine, Kanagawa, 216-8511 Japan; 3 Graduate School of Pharmacogenomics, St. Marianna University School of Medicine, Kanagawa, 216-8511 Japan

**Keywords:** Brain metastasis, Gynecological malignancies, Alpha-enolase, Triosephosphate isomerase, Transgelin-2

## Abstract

Brain metastases of gynecological malignancies are rare, but the incidence is increasing. Patients with brain metastases have a poor prognosis, therefore early detection and optimal management is necessary. In order to determine a new biomarker, we aimed to identify proteins that associated with brain metastases. We investigated proteins associated with brain metastases of gynecological malignancies in three patients who underwent surgical resection (stage IIb cervical cancer, stage Ib endometrial cancer, and stage IIIb ovarian cancer). Proteomic analysis was performed on formalin-fixed paraffin-embedded (FFPE) samples of the primary tumors and brain metastases, which were analyzed by liquid chromatography with tandem mass spectrometry. Thereafter, candidate proteins were identified by the Scaffold system and Mascot search program, and were analyzed using western blotting and immunohistochemistry. As a result, a total of 129 proteins were identified. In endometrial and ovarian cancers, western blotting revealed that the expression of alpha-enolase (ENO1) and triosephosphate isomerase (TPI-1) was higher and the expression of Transgelin-2 (TAGLN2) was lower in metastatic tumors than in primary tumors. On the other hand, the expression of TPI-1 and TAGLN2 was lower in metastatic tumors than in primary tumors in cervical cancer. Immunohistochemistry confirmed that ENO1 expression was elevated in the metastatic tumors compared with the primary tumors. In conclusion, the present study showed that FFPE tissue-based proteomics analysis can be powerful tool, and these findings suggested that ENO1, TPI-1, and TAGLN2 may have a role in the development and progression of brain metastasis from gynecological malignancies.

## Introduction

The incidence rate of gynecological malignancies is increasing in Japan, but mortality is stable due to more effective treatment and better diagnostic techniques. However, the treatment of advanced cancer remains problematic [1]. Metastasis is a major cause of morbidity and mortality in cancer patients, so investigation of the mechanisms involved is very important. Metastasis of cancer cells is a highly selective and non-random process that comprises a series of linked events. Various molecular and genetic changes define the multistep process of tumor dissemination, which has been described as the “metastatic cascade” [2]. Hematogenous metastasis is a complex biological process that includes the steps of intravasation, transport in the blood, extravasation, and growth in a distant organ [3]. For hematogenous metastasis to occur, every step of the cascade must be completed [4]. According to the “seed and soil” theory of Paget, organ selectivity of metastasis is based on the interaction between tumor cells (the “seed”) and the microenvironment of the target organ (the “soil”), which supports extravasation, survival, and growth of the metastatic tumor [5]. Metastasis of gynecological malignancies can occur via the lymphatic, hematogenous, and transcoelomic routes. For cervical cancer, the most frequent sites of metastasis are the lungs, paraaortic lymph nodes, supraclavicular lymph nodes, and abdominal cavity [6]. For endometrial cancer, the most frequent sites are the lung and liver, followed by other sites such as the adrenal gland, breast, bone, skin, and brain [7]. In the case of ovarian cancer, transcoelomic metastasis is the most common, followed by pelvic lymph node, peritoneal, lymphatic, and, rarely, by hematogenous spread [8]. Metastasis to the brain is one of the most feared complications of cancer, since patients with brain metastasis usually have a poor prognosis and rapidly progressive neurologic symptoms. Consequently, treatment of brain metastases is becoming an increasingly important determinant of the survival time and quality of life for cancer patients, meaning that early detection and optimal management of brain metastases are essential. According to the brain tumor registry of Japan (1984–1996), tumors of the lung (52.3 %), breast (8.9 %), and rectum (5.2 %) are most likely to metastasize to the brain, while brain metastasis from gynecological malignancies is rare (1.7 % for uterine cancer and 0.8 % for ovarian cancer) [9]. However, a recent study suggested that the incidence of brain metastasis from gynecological malignancies is rising along with the longer survival of patients with these tumors due to effective treatment and the availability of better imaging techniques [10].

DNA microarray analysis has now become a standard tool for molecular studies of cancer [11]. The ability to complement this approach with methods of proteomic analysis [12] is crucial for identification of proteins that may serve as targets for new antibody-based therapeutic strategies [13]. Such proteomic research is particularly important for the characterization of gene products contributing to the metastatic potential of cancer [14]. Molecular screening of metastases by proteomic analysis has been done in several previous studies, including investigations of breast cancer, hepatocellular carcinoma, and squamous cell carcinoma of the lung [15–17]. However, there has been little proteomic analysis of clinical samples of gynecological malignancies, including brain metastases.

Therefore, the present study was performed to investigate differential protein expression in patients with brain metastases of gynecological malignancies using proteomic analysis with the hope of identifying potential new tumor markers. To do this, we performed a comparative proteomic analysis of primary and metastatic tumor tissue sample from patients with gynecological cancer by liquid chromatography with tandem mass spectrometry (LC–MS/MS).

## Materials and methods

Proteomic analysis was performed on formalin-fixed paraffin-embedded samples of primary tumors and brain metastases, which were analyzed by LC–MS/MS. Candidate proteins were detected using the Scaffold system and Mascot search program. The expression of some proteins was also assessed by western blotting and immunohistochemistry.

### Patients

We reviewed 15 patients with brain metastases of gynecological malignancies. Between 2005 and 2009, 3 of them underwent surgical resection, including 1 patient each with uterine cervical cancer (FIGO stage IIb), endometrial cancer (FIGO stage Ib), and ovarian cancer (FIGO stage IIIb) (Table 1). Tumor tissue samples were fixed in 10 % buffered formalin for 24–48 h and then were embedded in paraffin, after which blocks of these specimens were stored from 2005 to 2009. The study protocol was approved by the Human Ethics Review Committee of St. Marianna University School of Medicine.

**Table 1:** Clinical characteristics of the patients

	Patients		
	1	2	3
Diagnosis	EC	CeC	OC
FIGO stage	Ib	IIb	IIIb
Histology	Endometrioid adenocarcinoma, G1	Squamous cell carcinoma	Serous adenocarcinoma
Primary treatment	Modified radical hysterectomy + BSO + PLN + chemotherapy	Radical hysterectomy + CCRT	Total hysterectomy + BSO + omentectomy + chemotherapy
Number of brain metastases	1	1	1
Treatment of brain metastasis	Surgery + WBRT	Surgery + WBRT	Surgery + WBRT
Other sites of disease	None	LN (paraaortic, Virchow’s) pulmonary	Abdominal
Survival^a^ (months)	AWD (48)	DOD (22)	DOD (4)

*EC* endometrial cancer, *CeC* uterine cervical cancer, *OC* ovarian cancer, *CCRT* concurrent chemoradiotherapy, *BSO* bilatelal salpingo-oophorectomy, *PLN* pelvic lymph node dissection, *LN* lymph node, *WBRT* whole brain radiotherapy, *AWD* alive with disease, *DOD* dead of disease

^a^Survival from diagnosis of brain metastasis

### Extraction of proteins from tumor tissues

To minimize contamination of samples by stromal cells, we selected the block that contained the largest amount of tumor tissue from each patient. Extraction of crude proteins from these tissue blocks was carried out as described elsewhere [18], with minor modifications. Briefly, 10 tissue sections (each 10 μm thick) were deparaffinized in 1 mL of xylene with gentle agitation for 5 min. After removing the xylene, 1 mL of 100 % ethanol was added and the sections were agitated for 5 min. After centrifugation at 15,000*g* for 10 min, the supernatant was removed and the pellet was thoroughly dried under a vacuum for 10 min. Then, 100 μl of extraction buffer from the Qproteome FFPE Tissue Kit (Qiagen, Valencia, CA, USA) was added to the dewaxed tissue pellet, followed by incubation on ice for 5 min, vortex mixing, and heating at 100 °C for 20 min and 80 °C for 2 h in a Thermomixer at 750 rpm. After centrifugation at 14,000*g* and 4 °C for 15 min, 10 μL of the supernatant was used to measure the protein content by the Lowry method [19].

### Sample preparation and LC–MS/MS

For isolation of tissues and preparation of proteins, the Qproteome FFPE Tissue Kit (Qiagen) was employed according to the manufacturer’s recommendations. After the total protein content was measured by the Lowry method, protein samples (50 μg) were divided up for LC–MS/MS analysis. These protein samples were digested with trypsin (Protease MAX Surfactant; Promega, Madison, WI, USA) and then extracted with a Zip tip C_18_ pipette tip (Promega).

The resulting peptides were subjected to LC–MS/MS analysis using a capillary LC system (Magic2002; Michrom BioResources, Auburn, CA, USA) coupled to an inline nanoelectrospraymass spectrometer (LCQAdvantage; Thermo Finnegan, Waltham, MA, USA) with a silica-coated glass capillary tube (PiclTip; New Objective, Woburn, MA, USA) to obtain a peptide mass fingerprint. Raw LC–MS/MS data files were searched by both Mascot and X!Tandem for identification. To generate a statistically valid list of proteins, Scaffold was used to accommodate differences of algorithm and score calculation by the two search engines [20]. Each protein identified was assigned a biological process based on information from the international protein index (IPI) human database (European Bioinformatics Institute, 2011) and the Gene Ontology (GO) database (National Center for Biotechnology Information, 2011).

### Western blotting

Protein samples (10 μg each) were mixed in sample buffer at 100 °C for 5 min and subjected to SDS-polyacrylamide gel electrophoresis on 10 % polyacrylamide gels. Then, the samples were transferred to enhanced chemiluminescence membranes (ECL; Amersham-Pharmacia Biotech, Buckinghamshire, UK) that had been blocked for 1 h in ECL Advance Blocking Agent. These membranes were incubated with rabbit anti–transgelin-2 (TAGLN2) polyclonal antibody (1:200; Sigma, Saint Louis, MO, USA), rabbit anti–triosephosphate isomerase 1 (TPI-1) polyclonal antibody (1:200; GeneTex, Irvine, CA, USA), or rabbit anti–enolase 1 (ENO1) polyclonal antibody (1:250; Abcam, Cambridge, UK) for 1 h at room temperature. After washing for a total of 30 min with 5 exchanges of Tris buffered saline with Tween 20 (TBS-T), the membranes were incubated with peroxidase-labeled immunoglobulin G of the appropriate species for 1 h at room temperature. After washing a further 5 times with TBS-T, immunoreactive proteins were detected with an ECL Advance Western Blotting Detection System kit (Amersham-Pharmacia Biotech, Piscataway, NJ, USA) and an LAS-3000(Fujifilm, Tokyo, Japan). After stripping, the membranes were reprobed with mouse anti-actin monoclonal antibody (1:2,000; Sigma) as a loading control.

### Immunohistochemistry

Tissue blocks of the primary tumors and brain metastases were cut into 3-μm sections, which were dewaxed, rehydrated, and incubated with 3 % hydrogen peroxide for 5 min to block endogenous peroxidases. Then, the sections were incubated with anti-ENO1 monoclonal antibody (1:100; Abcam) at room temperature for 1 h. After washing 3 times with phosphate-buffered saline, the sections were incubated with a horseradish peroxidase-labeled polymer-conjugated anti-mouse secondary antibody (ENVISION + ; Dako, Copenhagen, Denmark) for 30 min at room temperature. Finally, color was developed with 3,3′-diaminobenzidine tetrahydrochloride.

## Results

### Proteins identified in the primary and metastatic tumors

**Fig. 1:**
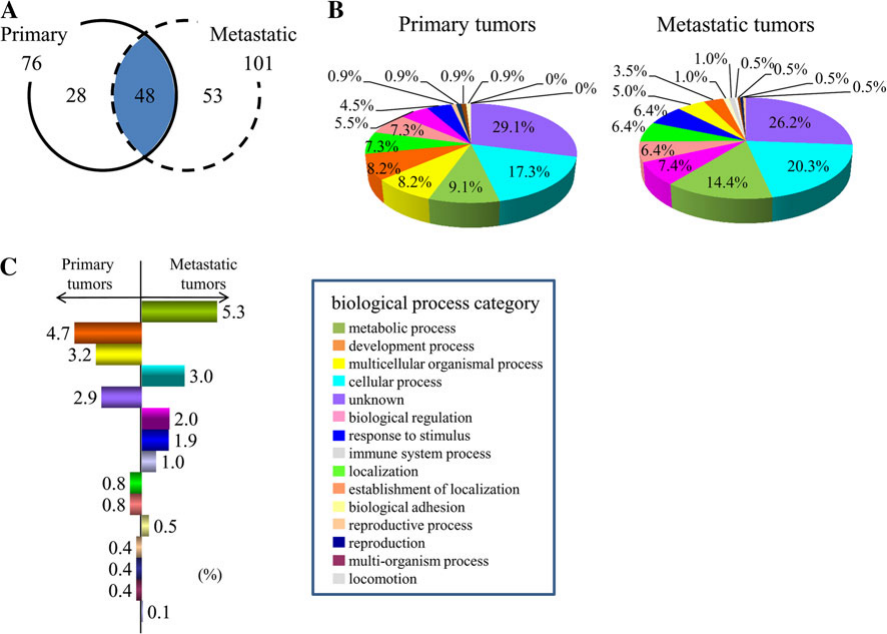
Protein expression in primary and metastatic tumors. **a** Venn diagram showing the differential expression of 129 proteins by primary and metastatic tumors (76 in primary tumors and 101 in metastatic tumors). **b** Distribution of proteins related to different biological processes. **c** Comparison of proteins expressed by primary and metastatic tumors. The expression of proteins related to developmental and multicellular organismal process was lower in metastatic tumors than in primary tumors, whereas the expression of proteins related to metabolic processes was higher in metastatic tumors than in primary tumors

Raw files of LC–MS/MS data were searched by both Mascot and X!Tandem for identification of proteins. To generate a statistically valid protein list, Scaffold was used to accommodate differences of algorithm and score calculation between the two search engines [20]. A total of 129 proteins were identified (76 in the primary tumors and 101 in the metastatic tumors) (Table 2). Comparison of the primary and metastatic tumors revealed the differential expression of 81 proteins (28 in the solely primary tumors and 53 in the solely metastatic tumors) and the shared expression of 48 other proteins (Fig. 1a).

**Table 2:** List of total expressed protein (protein identification probability)

No.	Protein name	Observed sample (probability %)					
		Primary tumor			Metastatic tumor		
		EC	CeC	OC	EC	CeC	OC
1	Actin, cytoplasmic 1	100	100	100	100	100	100
2	Histone H4	100	100	100	100	100	100
3	Keratin, type II cytoskeletal 1	100	90	100	100	100	100
4	Hemoglobin subunit beta	100	100	100	89	100	100
5	Histone H1.2	100	100	89	100	100	
6	Uncharacterized protein	93	100	98	100		100
7	Hemoglobin subunit alpha	93	100	100	89	100	89
8	Histone H2A type 1-B/E	100	100	89	100	100	89
9	Tubulin, beta			100	100	100	100
10	Keratin, type I cytoskeletal 10	93	100		100	100	89
11	Glyceraldehyde-3-phosphate dehydrogenase	100	90	100	100	100	100
12	Histone H3.2	100	90	89	94	100	89
13	Histone H2B type 1-L	98	99	89	89	99	
14	Isoform alpha-enolase of Alpha-enolase			100	100	100	100
15	Triosephosphate isomerase isoform 2	99			100	100	98
16	Vimentin			100		90	100
17	IGL@ protein		100		89	90	89
18	TUBA1C protein			89		100	98
19	Keratin, type I cytoskeletal 9	100			99		
20	Beta-actin-like protein 2		90	89		90	89
21	Neutrophil defensin 1	93	90		100	90	
22	Isoform M1 of Pyruvate kinase isozymes M1/M2		90			100	98
23	Heat shock protein beta-1		100	89		90	
24	Ubiquitin-40S ribosomal protein S27a		90		99		
25	Nuclease-sensitive element-binding protein 1	93	90		89		89
26	23 kDa protein	93			89	90	89
27	Keratin, type I cytoskeletal 19	93			89	90	89
28	Collagen alpha-2(I) chain	93	90	89	89		
29	Protein S100-A8	100	90			90	
30	Heat shock 70 kDa protein 1A/1B			89	89	100	
31	Protein S100-A9		100		100		
32	Isoform 1 of Fibronectin	93	90		89		
33	Galectin-1	93	90		89		
34	Isoform 1 of L-lactate dehydrogenase A chain	93			89	90	
35	PRO2275		90	89	89		
36	60S ribosomal protein L8	43	90			90	
37	N-acetyltransferase ESCO2	89			35	90	
38	cDNA FLJ45139 fis, clone BRAWH3039623		88		30	90	
39	40S ribosomal protein S25	93				90	
40	Isoform 1 of Heterogeneous nuclear ribonucleoprotein K	93				90	
41	Uncharacterized protein	93					89
42	14-3-3 protein theta	93			89		
43	Putative annexin A2-like protein	93			89		
44	Peptidyl-prolyl cis–trans isomerase A	93			89		
45	Fructose-bisphosphate aldolase A	93			89		
46	Glutathione S-transferase P		90			90	
47	Profilin-1			89			89
48	Histone H2A type 2-B		86				89
49	Isoform B1 of Heterogeneous nuclear ribonucleoproteins A2/B1				100	90	73
50	Isoform 1 of Glial fibrillary acidic protein					90	100
51	Ubiquitin-like modifier-activating enzyme 1						100
52	Elongation factor 1-alpha 1					100	
53	Beta-2-microglobulin					90	89
54	14-3-3 protein zeta/delta					90	89
55	Isoform Long of 14-3-3 protein beta/alpha				89	90	
56	ATP synthase subunit alpha, mitochondrial				89	90	
57	Putative uncharacterized protein				89	90	
58	Fibrinogen beta chain				100		
59	Heat shock protein HSP 90-beta						100
60	Keratin, type I cytoskeletal 18						100
61	Isoform 1 of heterogeneous nuclear ribonucleoprotein A3					100	
62	Stress-70 protein, mitochondrial						100
63	Keratin, type II cytoskeletal 8						100
64	ATP synthase subunit beta, mitochondrial						100
65	Peroxiredoxin-1					90	
66	Histone H1x					90	
67	60S ribosomal protein L7a					90	
68	Isoform short of heterogeneous nuclear ribonucleoprotein U					90	
69	Isoform 3 of probable ATP-dependent RNA helicase DDX17					90	
70	Homeobox protein HMX3					90	
71	Isoform 1 of tropomyosin alpha-4 chain						89
72	Isocitrate dehydrogenase [NADP], mitochondrial						89
73	Poly(rC)-binding protein 1						89
74	Thioredoxin-dependent peroxide reductase, mitochondrial						89
75	NHP2-like protein 1						89
76	Phosphoglycerate kinase 1						89
77	Elongation factor 2						89
78	Thioredoxin						89
79	Phosphoglycerate mutase 2						89
80	Isoform 1 of clusterin						89
81	Isoform mitochondrial of fumarate hydratase, mitochondrial						89
82	Isoform 2 of protein disulfide-isomerase A6						89
83	Isoform 1 of 3,2-trans-enoyl-CoA isomerase, mitochondrial						89
84	Isoform 2 of heterogeneous nuclear ribonucleoprotein A/B						89
85	Keratin, type II cytoskeletal 1b						89
86	32 kDa protein						89
87	Hemoglobin subunit delta						89
88	14 kDa protein						89
89	Isoform 1 of heat shock cognate 71 kDa protein				89		
90	Histone H2B type 2-E				89		
91	Plasminogen				89		
92	Keratin, type II cytoskeletal 2 epidermal				89		
93	Isoform 1 of heterogeneous nuclear ribonucleoprotein D0				89		
94	ADP-ribosylation factor 1				89		
95	Isoform A1-B of heterogeneous nuclear ribonucleoprotein A1				89		
96	Isoform C1 of heterogeneous nuclear ribonucleoproteins C1/C2				89		
97	Isoform 1 of myelin proteolipid protein				89		
98	14-3-3 protein gamma				89		
99	Isoform 1 of brain acid soluble protein 1				89		
100	cDNA FLJ35730 fis, clone TESTI2003131, highly similar to ALPHA-1-ANTICHYMOTRYPSIN				89		
101	Isoform 2 of Nucleophosminisoform 2 of nucleophosmin				89		
102	Myosin light chain 6B	93	90	89			
103	60S ribosomal protein L7	93	99				
104	Histone H1.5	93	98				
105	Transgelin		100				
106	Phosphatidylethanolamine-binding protein 1	93	90				
107	Transgelin-2		90	89			
108	Isoform long of splicing factor, proline- and glutamine-rich	93					
109	40S ribosomal protein S14	93					
110	Myosin regulatory light chain 12B	93					
111	40S ribosomal protein S13	93					
112	Isoform 1 of Protein shisa-6 homolog	93					
113	Histone H2A.V		90				
114	Lumican		90				
115	Prolargin		90				
116	HLA class I histocompatibility antigen, Cw-1 alpha chain		90				
117	Isoform 2 of microtubule-actin cross-linking factor 1, isoforms 1/2/3/5		90				
118	Macrophage migration inhibitory factor		90				
119	Isoform 1 of FYVE, RhoGEF and PH domain-containing protein 5		90				
120	31 kDa protein		90				
121	Centrosomal protein 170 kDa		90				
122	Isoform 1 of DNA polymerase theta		90				
123	Isoform 1 of serine/arginine-rich splicing factor 7			89			
124	Heterogeneous nuclear ribonucleoprotein H			89			
125	Hypothetical protein LOC80164			89			
126	Acyl-CoA-binding domain-containing protein 7-like			89			
127	NOL1/NOP2/Sun domain family member 4			89			
128	Actin, alpha skeletal muscle		90				
129	60S ribosomal protein L31	93					

Protein expression profiles of metastatic and primary tumor determined by LS/MS/MS. List of proteins found differentially expression between brain metastatic tumor and primary tumor. Protein identification probability is shown by the percentage of total spectra

*EC* Endometrial cancer,* CeC* uterine cervical cancer, *OC* ovarian cancer

### Proteomes of the primary and metastatic tumors

Each protein that we identified was assigned a biological process based on information from the IPI and GO databases to understand their role. If a protein was known to participate in more than one biological process, it was included in multiple categories. Classification according to biological processes showed that the majority of the proteins were involved in metabolic processes, developmental processes, or multicellular organismal process (Fig. 1b), with some interesting proteins that have been implicated in tumorigenesis. In metastatic tumors, proteins related to developmental and multicellular organismal process were decreased compared with primary tumors (4.7 and 3.2 %, respectively), while proteins related to metabolic processes were increased compared with primary tumors (5.3 %) (Fig. 1c).

### Differential protein expression by primary and metastatic tumors

Comparison of the distribution of proteins between primary and metastatic tumors revealed differences of proteins involved in metabolic, developmental, and multicellular organismal processes (Fig. 1c). Based on the results of searches carried out in the primary and metastatic tumors, eight candidate proteins were selected (Table 3). Among these proteins, TAGLN2, TPI-1, and ENO1 were subjected to further investigation.

**Table 3:** Protein expression profile of the primary and metastatic tumors

Protein category	Gene	p*I*	MW	Observed sample (Mascot score)					
				Primary tumors			Metastatic tumors		
				EC	CeC	OC	EC	CeC	OC
Metabolic process									
Triosephosphate isomerase 1	TPI1	5.7	30.8	+(111)		–	+(200)	+(96)	+(60)
Alpha-enolase	ENO1	7.0	47.1	–	–	+(112)	+(206)	+(94)	+(28)
ATP synthase subunit alpha, mitochondrial	ATP5A	9.2	59.7	–	–	–	+(62)	+(72)	–
Tubulin beta	TUBB	7.8	47.0	–	–	+(58)	+(96)	+(124)	+(97)
60S ribosomal protein L7	RPL7	10.7	29.2	+(63)	+(59)	–	–	–	–
Developmental precess									
Myosin light chain 6B	MYL6	5.6	22.8	+(52)	–	+(75)	–	–	–
Transgelin-2	TAGLN2	8.4	22.4	–	+(66)	+(86)	–	–	–
Multicellular organismal process									
Phosphatidylethanolamine-binding protein 1	PEBP1	8.6	26.4	+(40)	+(35)	–	–	–	–

Probabilities of 95 and 80 % were used as the cutoff values for identification of peptides and proteins, respectively, excluding proteins identified with a lesser probability

*EC* Endometrial cancer,* CeC* uterine cervical cancer, *OC* ovarian cancer

### Confirmation of differential protein expression

**Fig. 2:**
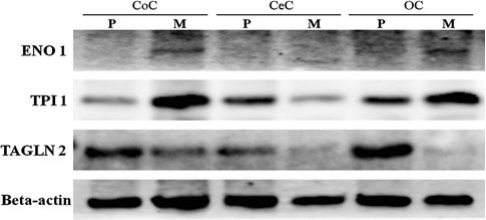
Western blot analysis of ENO1, TPI-1, and TAGLN2. Proteins from primary and metastatic tumors were separated by SDS-PAGE and transferred to PVDF membranes, followed by detection using the respective primary antibodies and an HRP-conjugated secondary antibody. In endometrial and ovarian cancers, the expression of ENO1 and TPI-1 was higher in metastatic tumors than in primary tumors, and the expression of TAGLN2 was lower in metastatic tumors than in primary tumors. Further, in cervical cancer, the expression of TPI-1 and TAGLN2 was lower in metastatic tumors than in primary tumors. *P* primary tumors, *M* metastatic tumors

To more precisely evaluate TAGLN2, TPI-1, and ENO1 expression by gynecological cancers, western blotting was carried out using proteins extracted from the 3 primary and 3 metastatic tumors. In endometrial and ovarian cancers, the expression of TPI-1 and ENO1 was higher in metastatic tumors than in primary tumors; this finding was consistent with the reported role of these proteins in promoting tumor cell survival and proliferation [21, 22]. However, the expression of TAGLN2 was lower in metastatic tumors than in primary tumors; this finding was consistent with the reported role of TAGLN2 as a tumor suppressor [23]. Further, in cervical cancer, the expression of TAGLN2 and TPI-1 was lower in metastatic tumors than in primary tumors (Fig. 2).

**Fig. 3:**
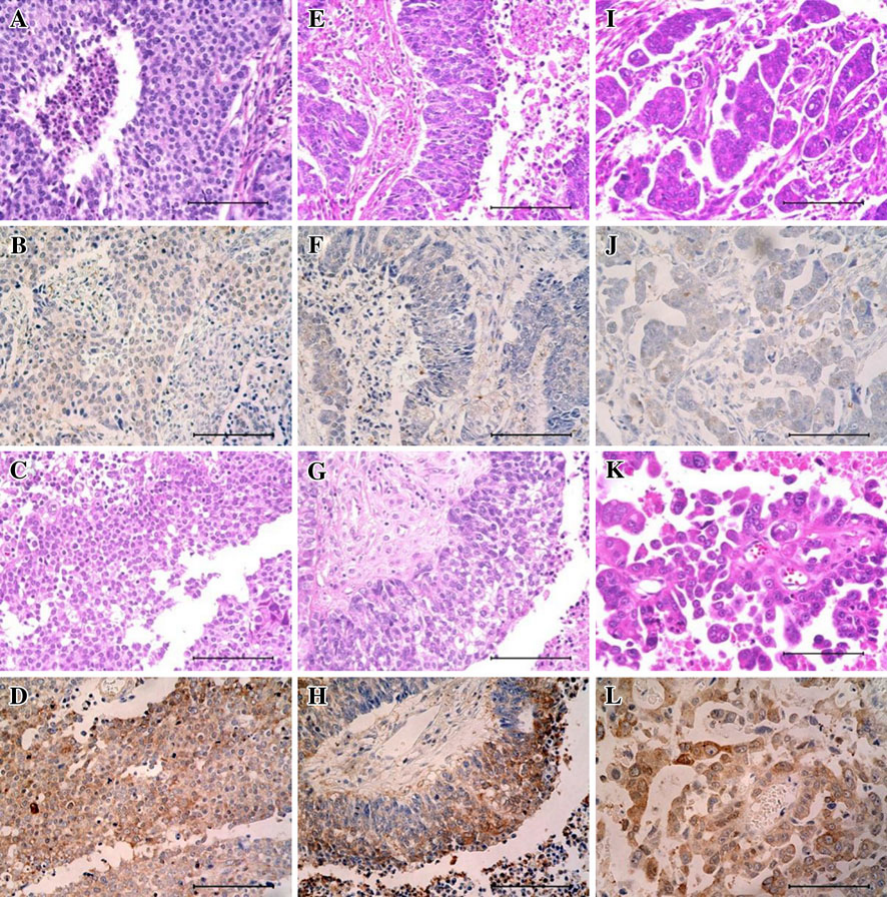
Representative immunostaining of ENO1 in primary and metastatic tumors. **a**, **b**, **e**, **f**, **i**, and **j** show primary tumors. **c**, **d**, **g**, **h**, **k**, and **l** show metastatic tumors. **a**, **e**, **i**, **c**, **g**, and **k** show HE staining. **b**, **f**, **j**, **d**, **h**, and **l** show ENO1 staining. ENO1 is overexpressed in metastatic tumors. All images are ×400 magnification. *Scale bars* 100 mm. *EC* endometrial cancer (**a**–**d**). *CeC* Uterine cervical cancer (**e**–**h**). *OC* ovarian cancer (**i**–**l**)

In addition, immunohistochemical analysis was employed to assess ENO1. Figure 3 shows representative examples of immunostaining for ENO1 in primary and metastatic tumors. Both western blotting and immunohistochemistry confirmed the findings of LC–MS/MS analysis.

## Discussion

The aim of this study was to identify biomarkers for brain metastases of gynecological malignancies, which have a different protein expression profile compared with primary tumors. Identification of proteins that are up-regulated or down-regulated in metastatic tumors may facilitate the detection and/or treatment of metastasis cancer. Therefore, we performed a comparative proteomic analysis of primary and metastatic gynecological cancers by LC–MS/MS analysis of proteins extracted from FFPE samples. Western blotting and immunohistochemistry were also performed to confirm the results of LC–MS/MS analysis. Until recently, protein extraction from formalin-fixed tissues was thought to be impossible because fixation by formalin creates strong intermolecular covalent bonds [24]. However, successful protein extraction protocols have been established based on the heat-induced antigen retrieval technique widely applied for immunohistochemistry and proteomic analysis by LC–MS/MS [25, 26]. In the present study, the protein expression profile of metastatic tumors was compared with that of primary tumors using extracts of FFPE samples. By LC–MS/MS and scaffold analysis, 76 proteins were identified in 3 primary tumors and 101 proteins were found in 3 metastatic tumors. These proteins were related to a variety of biological processes (Fig. 1b). To find candidate proteins, the 129 proteins that we identified were divided into 15 categories based on biological processes. Comparison of the distribution of these proteins between the primary and metastatic tumors showed differences in the expression of proteins related to metabolic, developmental, and multicellular organismal processes (Fig. 1c). Eight candidate proteins were selected that were predominantly or exclusively expressed by either the primary or metastatic tumors (Table 3). Several of the proteins identified in this study have been reported previously as possible metastasis-related proteins, including TAGLN2, TPI-1, ENO1, phosphatidylethanolamine binding protein (PEBP1), and mitochondrial ATP synthase alpha-subunit (ATP5A). TAGLN2 is a poorly characterized member of the calponin family. Its closest homologue is Transgelin 1/SM22a, an actin cross-linking protein [27] that is thought to undergo down-regulation as an early marker of transformation [28]. A recent study suggested that TAGLN2 has a negative influence on metastasis suppressing the invasive capacity of tumor cells [23]. TPI-1 is an enzyme that catalyzes the reversible transformation of d-3-glyceraldehyde phosphate into dihydroxyacetone phosphate. Dihydroxyacetone phosphate is then transformed into d-3-glyceraldehyde phosphate to continue the glycolytic pathway, so TPI-1 has an important role in the process of glycolysis [29]. Changes of enzyme activity have been reported under normal and pathological conditions, and overexpression of TPI-1 may activate both energy production and protein synthesis/degradation in rapidly growing tumor cells [30]. Another study suggested that metabolic changes are associated with markedly enhanced survival and proliferation of breast cancer in the brain metastasis [21]. ENO1 is a glycolytic enzyme that catalyzes the conversion of 2-phosphoglycerate into phosphoenolpyruvate [31]. ENO1 is the frequently deregulated in various types of cancer [32]. In addition, ENO1 is more highly expressed in metastatic cancer cells compared with primary cancer cells, suggesting an oncogenic role of ENO1 [22]. PEBP1 was originally identified as an endogenous inhibitor of Raf, and it negatively regulates the Raf/MEK/ERK-signaling cascade [33]. It has been well established that PEBP1 suppresses the metastatic spread of tumor cells, and, moreover, the down-regulated expression of PEBP1 is observed in a number of human cancers [34]. ATP5A was identified in tumor metastasized to liver and was overexpressed in metastatic tumor [35].

These reports indicated that there are several changes of protein expression between primary and metastatic tumor, and the metastasis may be supported by the expression changes. In the present study of clinical tumor samples, we identified the expression of TAGLN2 in primary tumors and we also identified TPI-1 and ENO1 in metastatic tumors, predominantly by LC–MS/MS. Although the three proteins identified in this study have previously been suggested to be cancer-related, their functional role in gynecological cancer remains controversial. Also, we investigated the three proteins by western blotting. There were a few disparities in the results between western blot and LC–MS/MS. We described that TPI-1 was predominantly identified in metastatic tumors. However, the TPI-1 expression in primary tumor was higher than metastatic tumor in cervical cancer. It has been reported that TPI-1 was represented overexpression in tumor cells [30]. In addition, the overexpression of TPI-1 in metastatic tumor may not be a necessary character for metastasis. In any case, difference of cancer type may indicate difference of metastatic character. Further study is necessary to obtain their credibility as biomarkers.

Various molecular and genetic changes occur during the multistep process of tumor dissemination, and these have been called the “metastatic cascade” [2]. This cascade starts with escape from the primary tumor by invasion of the surrounding tissue, entry into the bloodstream (intravasation), extravasation at a distant site, and finally survival and proliferation to form metastases [36, 37]. In this study, expression of TPI-1 and ENO1, which enhances cancer cell survival and proliferation, were higher in metastatic tumors than in primary tumors, and TAGLN2, which suppresses invasion of cancer cells, was lower in metastatic tumors than in primary tumors. The different expression of these proteins between primary and metastatic tumor may require characteristics for brain metastasis in these gynecological cancers. In addition, tubulin beta, 60S ribosomal protein L7 and Myosin light chain 6B were also identified in this study. Their role in tumor metastasis requires further investigation, but the role may be similar to that of proteins such as TPI-1, ENO1, and TAGLN2.

A recent study indicated that primary tumors can be regarded as genetically heterogeneous and contain subpopulations of cells with varying levels of metastatic potential [38]. The data indicate that primary tumors are subpopulations of cells with different characteristics. This study indicated, by difference of identified proteins, that cancer cells can have different characteristics between primary tumors and metastatic tumors. The different characteristics might relate to brain metastasis in this study.

In conclusion, we identified several proteins that may be involved in brain metastasis of gynecological malignancies. Since our study consisted of a small number of patients, a larger study should be conducted in order to define the biological functions and influence in tumorigenesis and metastasis. In this study, we demonstrated the biomarker analysis tool using FFPE tissue-based proteomics by comparison of expression protein. Future studies using this tool will contribute to analysis of biomarkers, not only brain metastasis but also other organ metastasis such as lung and lymph node.
